# Bioinformatic identification of genomic instability-associated lncRNAs signatures for improving the clinical outcome of cervical cancer by a prognostic model

**DOI:** 10.1038/s41598-021-00384-6

**Published:** 2021-10-22

**Authors:** Jian Zhang, Nan Ding, Yongxing He, Chengbin Tao, Zhongzhen Liang, Wenhu Xin, Qianyun Zhang, Fang Wang

**Affiliations:** 1 Department of Reproductive Medicine, Lanzhou University Second Hospital, Lanzhou, 730030 China; 2 School of Life Sciences, Lanzhou University, Lanzhou, 730000 China

**Keywords:** Cancer genetics, Cancer genomics, Genetics, Oncology

## Abstract

The research is executed to analyze the connection between genomic instability-associated long non-coding RNAs (lncRNAs) and the prognosis of cervical cancer patients. We set a prognostic model up and explored different risk groups' features. The clinical datasets and gene expression profiles of 307 patients have been downloaded from The Cancer Genome Atlas database. We established a prognostic model that combined somatic mutation profiles and lncRNA expression profiles in a tumor genome and identified 35 genomic instability-associated lncRNAs in cervical cancer as a case study. We then stratified patients into low-risk and high-risk groups and were further checked in multiple independent patient cohorts. Patients were separated into two sets: the testing set and the training set. The prognostic model was built using three genomic instability-associated lncRNAs (AC107464.2, MIR100HG, and AP001527.2). Patients in the training set were divided into the high-risk group with shorter overall survival and the low-risk group with longer overall survival (*p* < 0.001); in the meantime, similar comparable results were found in the testing set (*p* = 0.046), whole set (*p* < 0.001). There are also significant differences in patients with histological grades, FIGO stages, and different ages (*p* < 0.05). The prognostic model focused on genomic instability-associated lncRNAs could predict the prognosis of cervical cancer patients, paving the way for further research into the function and resource of lncRNAs, as well as a key approach to customizing individual care decision-making.

## Introduction

The major cause of cancer mortality among women around the globe is cervical cancer (CC) which ranks 4th as a widely diagnosed cancer. Early CC patients were tested with thinprep cytologic tests (TCT) and treated with human papilloma (HPV) vaccines, but mortality between 2007 and 2017 rose by 19%^1^. Particularly in developing countries, the long-term survival and prognosis of patients at advanced stage CC remain still poor. Patient features (such as age, the high-risk HPV infection, cancer grade, etc.) are already used to evaluate the recurrence or progression of patients with CC. CC is considered to be a complex, clinical heterogeneity cancer. Surgery, radiotherapy, and chemical treatment are often used for CC, but such treatments do not necessarily work^2^. Therefore, there is an evident interest in finding new bioinformatic identification and novel therapeutic targets, which are capable of could reliably predict the clinical outcomes of CC accurately.

Genomic instability was established by increasing the incidence of gene destruction and genomic integrity loss as a significant feature of tumorigenesis^3^. More importantly, genomic instability is correlated and a prognostic factor with tumor development and survival^4–6^. Though it is uncertain that disrupting the mechanism of genomic stability, numerous studies have confirmed that long noncoding RNA (lncRNA) is functional in such a process^3,7–9^.

In this study, we established a computational model integrating lncRNA expression profiles and somatic mutation profiles in a tumor genome to explore better the dynamic mechanism of lncRNA signature as an indicator of CC genomic stability, and which might help improve its prognostic utility.

## Materials and methods

### Data collection

The data were collected from The Cancer Genome Atlas (TCGA) database included clinical features, transcriptome profiling data, and somatic mutation information of CC patients. 307 female samples were paired with the Fragments Per Kilobase Million (FPKM) values of lncRNA and mRNA expression profiles, somatic mutation data, and clinical survival data were to further analyze and validate. Data were deposited in the TCGA database (https://portal.gdc.cancer.gov/repository).

The training set was used to identify prognostic lncRNA signature and build a prognostic risk model. The testing set was used to validate the efficiency of the prognostic risk model independently. Besides, somatic mutation information and the corresponding lncRNA expression data of 294 CC patients were also downloaded from the TCGA database. The clinical and pathological characteristics were briefly summarized in Table 1.

**Table 1:** Clinical information for 3 cervical cancer patients sets in this study.

Characteristics	Testing set (n = 152)	Training set (n = 152)	Whole set (n = 304)	*p*-value*
**Age, no (%)**				
Young (≤ 46)	76 (50)	78 (51.32)	154 (50.66)	0.9087
Old (> 46)	76 (50)	74 (48.68)	150 (49.34)	
**Histological grade, no (%)**				
G1–2	70 (46.05)	83 (54.61)	153 (50.33)	0.1087
G3	66 (43.42)	52 (34.21)	118 (38.82)	
Unknow	16 (10.52)	17 (11.18)	33 (10.86)	
**FIGO stage no (%)**				
Stage I–IIA	97 (63.81)	91 (59.87)	188 (61.84)	0.3421
Stage IIB–IVB	50 (32.89)	59 (34.21)	109 (35.86)	
Unknow	5 (3.29)	2 (1.32)	7 (2.30)	
**T, no (%)**				
T1–2	104 (68.42)	107 (70.39)	211 (69.41)	0.1492
T3–4	10 (6.58)	20 (13.16)	30 (9.87)	
Unknow	38 (25)	25 (16.45)	63 (20.72)	
**M, no (%)**				
M0	57 (37.5)	59 (38.82)	116 (38.16)	0.1494
M1	2 (1.32)	8 (5.26)	10 (3.29)	
Unknow	93 (61.18)	85 (55.92)	178 (58.55)	
**N, no (%)**				
N0	70 (46.05)	63 (41.45)	133 (43.75)	0.2982
N1	26 (17.11)	34 (22.37)	60 (19.74)	
Unknow	56 (36.84)	55 (36.18)	111 (36.51)	
**Vital status, no (%)**				
Alive	124 (81.58)	110 (72.37)	234 (76.97)	0.0766
Dead	28 (18.42)	42 (27.63)	70 (23.03)	

*Compared testing set with training set by using Chi square test.

### Identification of genomic instability-associated lncRNAs

**Figure 1:**
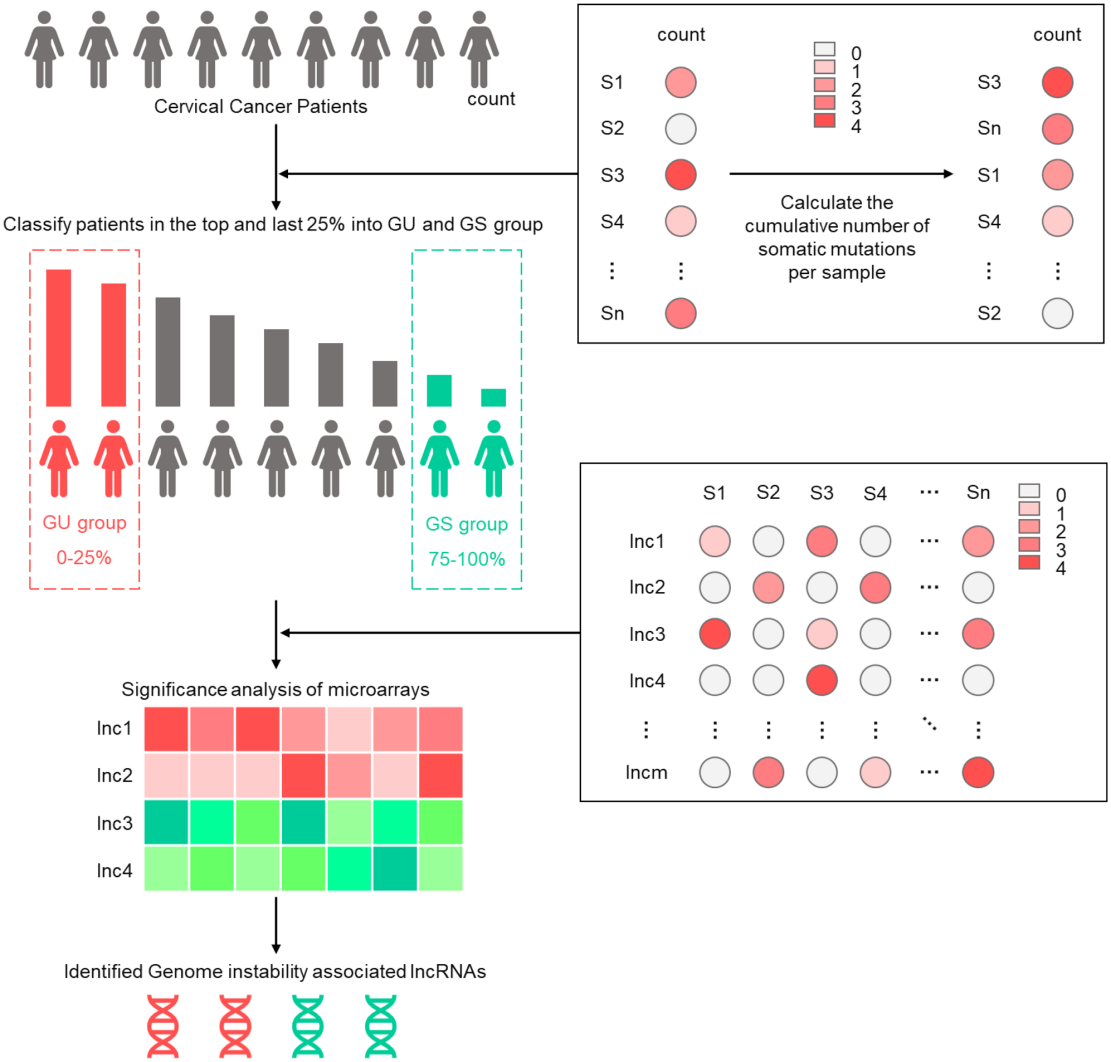
Computational process of genomic instability-related lncRNAs detection. Calculating the cumulative number of somatic mutations per sample and ranked in decreasing order. Then, somatic mutation profile was built. The columns reflect cervical cancer samples, and the rows reflect genes. The value reflects the number of altered sites for each gene on each sample. Samples were divided into two groups, GU-like group (patients’ mutator phenotype ranked in the top 25%) and GS-like group (the last 25%), according to their mutator phenotype. Genomic instability-related lncRNAs were detected by comparing the lncRNA expression profile between GU group and GS group. Differentially expressed lncRNAs were defined as genomic instability-associated lncRNAs.

Briefly, we followed the methods of Bao et al. 2019 to identify genomic instability-associated lncRNA and use a mutator hypothesis-derived computational model^10^. The computational model incorporating lncRNA expression profiles and somatic mutation profiles in a tumor genome to screen the genes that are significantly associated with lncRNAs (Fig. 1): (1) the cumulative number of somatic mutations was computed and ranked in decreasing order for each patient; (2) the top 25% of patients were defined as genomic unstable (GU)-like group, and the last 25% were defined genomically stable (GS)-like group; (3) expression profiles of lncRNAs between the GU group and GS group were compared using significance analysis of microarrays (SAM) method; (4) differentially expressed lncRNAs (|log fold change|> 0.3 and false discovery rate (FDR) adjusted *p* < 0.05) were defined as genomic instability-associated lncRNAs^11^.

### Establishment of the prognostic model and validation

For the construction of the prognostic model, CC patients with overall survival of < 30 days were excluded. To select prognostic genes, we applied Univariate Cox regression analysis by R package survival (https://github.com/therneau/survival) with a cut-off of *p* < 0.05. The whole data set was randomly separated into the training set and the testing set using R package caret (https://github.com/topepo/caret).

We evaluated outcome prediction by using a lncRNA signature (LncSig) formula as follows: $LncSig\;\left( {{\text{patient}}} \right) = \mathop \sum \limits_{i = 1}^{n} ceof\;\left( {lncRNA_{i} } \right)*expr\;\left( {lncRNA_{i} } \right)$. *LncSig *(*patient*) represents a prognostic risk score, *expr* (*lncRNA*_*i*_) is the expression level of the ith prognostic lncRNA for the patient. *coef* (*lncRNA*_*i*_) represents prognostic risk scores of the ith prognostic lncRNA, and *coef* was calculated by multivariate Cox analysis. Cox regression and stratified analysis were used in evaluating the link between LncSig and some important clinical factors. We determined the risk score for each study based on the expression of the outcome-related genes, the prognosis model coefficient, and patients' survival status. We calculated hazard ratio (HR) and 95% confidence interval (CI) by Cox analysis. The samples were consequently separated by the risk score median value of the low-risk or high-risk group. Finally, all statistical analyses were carried out by using R-version 4.0.2 (https://www.R-project.org). R package (survivalROC) and the time-dependent receiver operating characteristic (timeROC) curve were evaluated the prognostic performance of the model LncSig.

### Functional enrichment analysis

The functional enrichment analysis was conducted using the R package (clusterProfiler). We have conducted the Pearson correlation to determine 15 LncRNAs (co-expressed LncRNA-associated mRNA partners) to determine the link between paired lncRNAs expression and protein-coding genes (PCGs) in CC. To improve the reliability and credibility of the results, we employed the Gene Ontology (GO) Enrichment Analysis and the Kyoto Encyclopedia of Genes and Genomes (KEGG) pathway enrichment analysis, which targeted the co-expressed lncRNA-associated mRNA partners to further explore the potential functions and the molecular mechanism of lncRNAs based on the threshold with FDR < 0.05 and *p* < 0.05.

## Results

### Identification of genomic instability-related lncRNAs in cervical cancer patients

**Figure 2:**
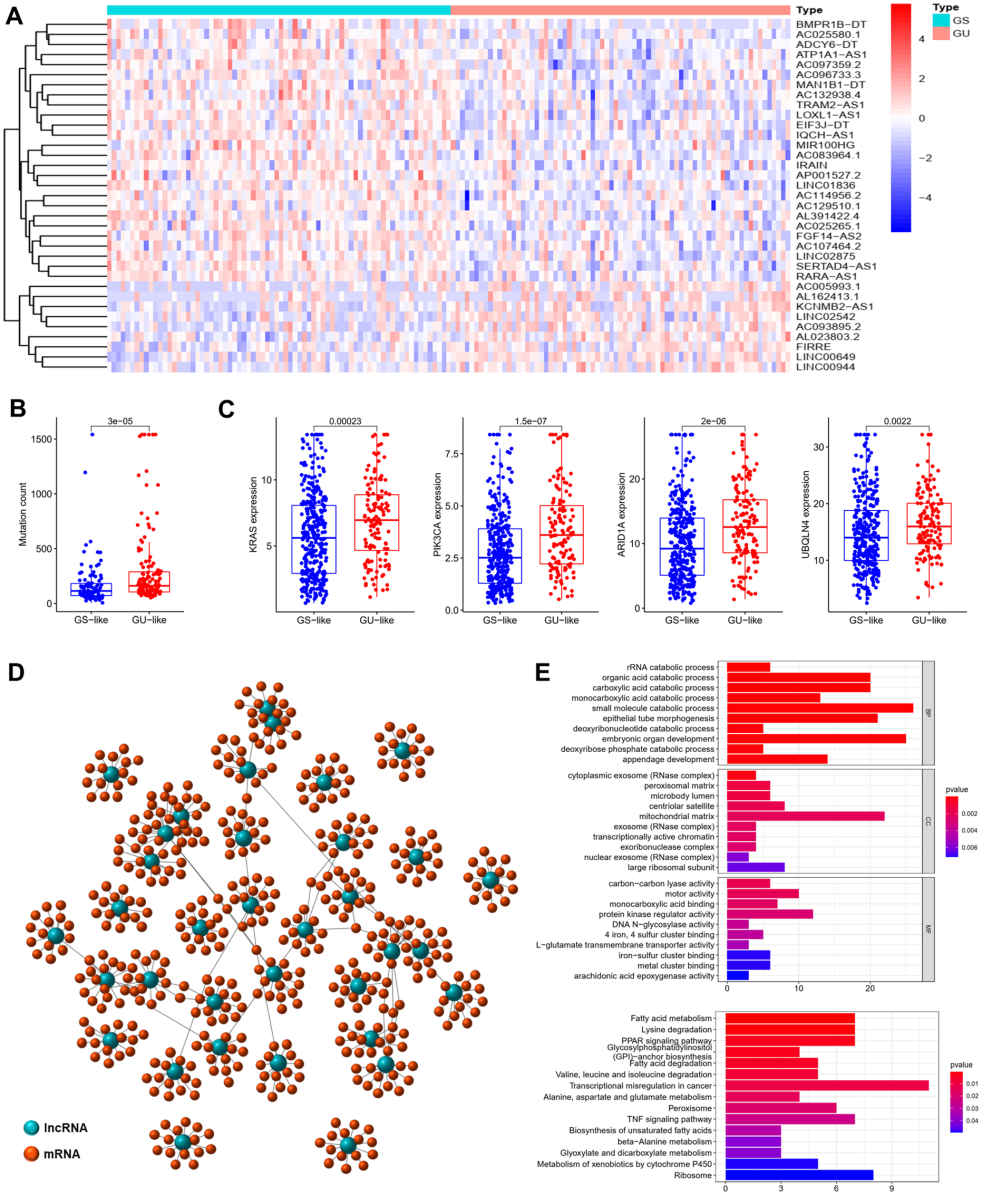
Identification and functional annotations of genomic instability-related lncRNAs in patients with cervical cancer. (**A**) Clustering of 147 cervical cancer patients based on the expression pattern of 35 candidate genomic instability-related lncRNAs. The left blue cluster is GS-like group, and the right red cluster is GU-like group. (**B**) Boxplots of somatic mutations in the GU-like group and GS-like group. Somatic cumulative mutations in the GU-like group are significantly higher than those in the GS-like group (*p* < 0.001). (**C**) Boxplots of KRAS, PIK3CA, ARID1A and UBQLN4 expression level in the GU-like group and GS-like group. These genes expression level in the GU-like group is significantly higher than that in the GS-like group (*p* < 0.001). (**D**) Co-expression network of genomic instability-related lncRNAs and mRNAs based on the Pearson correlation coefficient. The blue circles represent lncRNAs, and the red circles represent mRNAs. (**E**) Functional enrichment analysis of GO and KEGG for mRNAs co-expressed lncRNAs.

We collected 309 samples (306 tumor and 3 adjacent tissues) from the TCGA database to analyze the differences of gene expression between tumor and adjacent samples, and then identified the lncRNAs related to genomic instability in CC patients. The cumulative number of somatic mutations per patient was computed, and then ranked them in the decreasing order, the top 25% (n = 73) and last 25% (n = 74) as GU-like group and GS-like group according to the above order. 35 lncRNAs were found to be substantially differentially expressed with their |log fold change value|> 0.3 and FDR-adjusted *p* < 0.05 based on the SAM approach. We performed hierarchical clustering analysis on 147 samples of the whole set using the set of 35 differentially expressed lncRNAs, and then we clustered into GU and GS-like groups according to the expression levels of 35 differentially expressed lncRNAs (9 upregulated lncRNAs and 26 downregulated were found in GU-like group, R-package: limma, sparcl and pheatmap, Fig. 2A). Analytical findings revealed a statistically significant difference in the median value of somatic cumulative mutations between the GU-like (57.3) and the GS-like group (42.7), *p* < 0.001, Mann–Whitney U test, R-package: limma and ggpubr, Fig. 2B. We next compared the expression level of KRAS, PIK3CA, ARID1A, and UBQLN4 gene (a set of newly discovered drivers of genomic instability) between the GS-like group and GU-like group^12,13^. When compared to the GS-like group, the GU-like group showed greater these gene expression levels (*p* < 0.05, Mann–Whitney U test, R-package: limma and ggpubr, Fig. 2C).

We performed functional enrichment analysis to predict possible roles and pathways, and aim to further grasp the relationship between the expression of 35 differentially lncRNAs and PCGs. We calculated the expression correlation between the 35 lncRNAs and PCGs, and then found lncRNA-correlated PCGs. A network of lncRNAs–mRNA co-expression was built with 35 nodes, and one node containing 1 lncRNA and 15 mRNAs, and if they were related, the lncRNAs and mRNAs are connected (R-package: limma and igraph, Table 2, Fig. 2D). The results of GO analysis of lncRNA-correlated PCGs showed that mRNAs in this network were substantially linked with genomic instability, including rRNA catabolic process, deoxyribonucleotide catabolic process, and transcriptionally active chromatin (R-package: clusterProfiler, org.Hs.eg.db, enrichplot and ggplot2, Fig. 2E). KEGG pathway analysis identified 15 pathways that were highly enriched, several of which were associated with transcriptional misregulation in cancer (Fig. 2E). While analyzing the 35 differentially expressed lncRNAs, we found that their altered expression might affect transcriptional genes, which may cause the genomic stability in CC cells (Table 2). Normal gene damage repair boosts genomic instability due to changes in the cell microenvironment, and the genomic instability brought on by changes in the molecular and metabolism function of the lncRNA-related PCGs regulatory network. As shown in the above findings, and it was found that 35 lncRNAs whose expression differed from that of their normal tissues were potential genomic instability-associated lncRNAs (GIlncRNAs).

**Table 2:** Differentially expressed lncRNAs and relative mRNAs.

LncRNA	logFC	*p* value	Fdr	Relative mRNA
**Up-regulated**				
KCNMB2-AS1	1.633484	9.22E−05	0.018886	BOLA2B, LAMTOR4, UQCC3, BCL7C, FAAP20, NUDT1, IZUMO4, POLR2J, NCBP2AS2, COPS9, ELOB, NT5C, SMUG1, MRPL47, TNNC2
AC093895.2	1.544702	4.76E−05	0.014907	LPAR6, HEBP2, ARL11, C12orf54, CRABP2, HSPB1, NRN1, XCL1, SLC44A5, SP140L, KRT15, TXNDC17, PERM1, C1orf21, CLEC2A
AL162413.1	2.309286	0.001041	0.047346	KCNQ4, APRT, EXOSC4, CITED4, DPM2, PTGES2, RPL36, BOP1, RPL13, NDUFAF8, CCDC167, MRPL27, MRPL14, RPL8, MVD
FIRRE	0.974686	0.000931	0.046215	PAPOLG, TFAP4, KMT5A, KCTD15, KANSL2, KDM3A, METTL8, DKC1, TAF4B, ZC3H8, VANGL2, C21orf91, EFS, METAP1, FANCE
LINC00944	1.795497	0.000303	0.028837	SLC25A22, IL2RG, RELB, GNGT2, ICOS, IL21R, MARCO, FAM24B, APOBEC3G, APOBEC3H, CTLA4, CXCL10, VCAM1, CLIC2, CD2
AC005993.1	1.09773	0.000443	0.037799	TESMIN, ALPG, IGF1R, CCNA1, DDX17, CCDC3, ANO1, PREX1, DGKZ, KMT5B, SERHL2, TMEM184B, BRMS1L, MBIP, DNAL4
LINC02542	1.075473	0.000305	0.028837	A1CF, XYLB, GGCX, AGMAT, ACOX2, SLC25A13, ACADSB, SERPIND1, PLG, ITIH1, SLC6A1, AGMO, SLCO1B1, SNTB1, HNF4A
LINC00649	0.909561	1.81E−05	0.007423	IFI16, SNX30, N4BP1, GJB5, RARG, KCTD1, NECTIN1, MAP3K6, TRIM29, GM2A, KLF8, TRERF1, DEF6, NECTIN4, LRRC1
AL023803.2	0.553819	0.000561	0.037799	PAX9, CALB2, CCNO, FAM83D, MCIDAS, ITGA2B, UBE2C, LIN7B, FOXA1, PCED1A, AC011479.2, TFAP2C, MXD3, ACTR5, KMT5C
**Down-regulated**				
MAN1B1-DT	− 0.56543	0.000984	0.047346	EXOSC6, HIRIP3, DDX28, MDP1, CHAF1B, THAP11, TTC32, C4orf36, TLX2, C9orf78, CTF1, CFDP1, EXOSC2, PIGW, UTP4
AC025580.1	− 1.87066	0.000609	0.038385	TCTE3, SCIN, ZG16B, GFPT1, ZNF585B, FGFBP3, TTC39A, SLC44A4, ZNF345, MYO6, PDXDC1, ZFP14, ZNF529, ARFGEF3, ZNF518A
TRAM2-AS1	− 0.43872	0.000128	0.02102	ALDH5A1, BPHL, SIRT5, TPMT, KLC4, CAP2, ACOT13, MMUT, MOCS1, DHTKD1, HIBADH, YIPF3, SLC17A4, FAM8A1, EHHADH
RARA-AS1	− 0.35817	0.000119	0.02102	FKBP2, RARA, KRT18, TMEM205, RPS27L, NTHL1, G3BP1, REPS2, FUCA1, CEBPB, BLOC1S1, FAM167B, RAB17, COX14, CD63
LINC01836	− 0.99887	0.000317	0.028837	TMC4, MSLN, WWC1, MISP, RAB20, TMPRSS3, LAMA5, ALDH3B1, TSPAN15, DOCK5, RBMS2, CRIM1, IQCE, PIWIL4, CCL28
SERTAD4-AS1	− 0.88678	6.68E−06	0.005235	SERTAD4, DOK7, TMEM125, SIX1, CRIP2, HOXB6, HOXB5, CCDC160, MRAP2, TSPAN3, SLFN13, CRIP1, COL9A2, IFT172, SCX
AC132938.4	− 0.6378	7.33E−05	0.017157	PNPO, PIGV, CPT2, HLF, PDK2, TOM1L1, PCTP, SLC38A10, FBXO31, ACOX1, MTMR4, UGT1A3, SCP2, ZMYND12, CRYZ
AC107464.2	− 0.75617	0.001019	0.047346	PDE6B, UCP2, PRAF2, FUZ, DTX3, ZNF232, DOK1, AC005041.1, COL9A2, NAT14, CRIP1, UBXN11, C2orf15, C11orf49, CLUAP1
MIR100HG	− 0.8595	0.000184	0.025064	SPRY2, SPRY1, DLG4, KIF26B, MFGE8, ZNF853, FGF18, SPRY4, MFAP4, EFEMP2, REV3L, ETV5, VCAN, KCNH3, LRIG1
AC083964.1	− 0.47203	0.000799	0.042653	TDRP, CCDC28B, FABP6, MARCO, NPPC, KREMEN2, TNNI2, IL11RA, COL16A1, LIFR, FAM71E1, PARM1, CD200, TRAF2, SOCS1
IRAIN	− 0.1848	0.000699	0.042435	TESMIN, IGF1R, ALPG, CCNA1, CCDC3, ANO1, CSPG5, PREX1, TMEM184B, SLC39A8, RGS10, DNAL4, KMT5B, RNF32, DDX17
AP001527.2	− 1.66464	0.000317	0.028837	YAP1, BIRC2, CEP126, TMEM123, CFAP300, SYDE1, SLC1A6, DYNC2H1, DCUN1D5, FADS3, BIRC3, IKBIP, HMGB3, ELOVL3, GPAT2
BMPR1B-DT	− 2.95927	0.000575	0.037799	BMPR1B, SOX17, FBLN1, PAK1IP1, FAM189A2, MAP2K6, HOXA10, TUBA3D, RBBP7, AADAT, LHX2, ELP3, ASRGL1, IGF1, ALKAL2
AC096733.3	− 0.41954	0.000561	0.037799	TBC1D9, WDFY3, HELQ, USP53, ELF2, SMARCAD1, NEK1, KIAA1109, THUMPD1, SETD1B, KDM6A, KIDINS220, DNAJB14, ARID2, EIF2AK3
AC097359.2	− 0.38644	0.000777	0.042653	TCTA, SLC25A20, QPRT, TK2, FN3K, ABHD6, CMTM8, MYRIP, SLC26A1, ALDH4A1, CPN2, SYPL2, HNF1A, IQSEC1, OAF
ADCY6-DT	− 0.96724	0.000577	0.037799	JSRP1, PLA2G10, SMIM22, TRIM54, GCNT3, ASPHD1, PDE4C, METTL27, TNNC2, PRR13, RNASEH2C, PGP, RASSF7, ELOB, TMEM238
IQCH-AS1	− 0.40448	0.000238	0.027863	NEK8, NEIL1, C2orf15, COA5, DIS3L, P4HTM, SNAPC5, ZNF33B, BBS4, MYO5C, LZTS3, FAM81A, ARPIN, LRTOMT, CCDC57
AC129510.1	− 0.47954	0.000522	0.037799	CCDC14, AHI1, WDR90, NKTR, PHF12, PNISR, CFAP44, SREK1, MSANTD2, EFHC1, KIF27, VEZF1, PASK, DNAL1, KIAA0753
LINC02875	− 0.69396	0.000465	0.037799	PIGP, RAB6B, SOX2, C6orf226, CDKAL1, TNRC6C, TBX2, TMEM251, CHAF1B, CHST7, ADRA2B, TP53I13, BFSP1, CD200, THAP7
LOXL1-AS1	− 0.59163	9.59E−06	0.005235	LOXL1, ADPGK, CHSY1, LARP6, SLC35E4, RCN2, THAP10, KIAA0753, NCBP3, VCL, CHD3, DTX3, PTPN9, CNTROB, MYO9A
FGF14-AS2	− 1.20708	3.01E−06	0.004925	CMBL, ACAA2, TMEM205, BTD, CYP2B6, ZG16, CYP2A6, CYB5A, SERPINA4, HAO1, ACBD4, CLYBL, SLC10A1, CYP2A13, PCK2
AL391422.4	− 0.56864	0.000766	0.042653	PXDC1, TMEM14C, SAA2, CUTA, YIPF3, TRIM27, RNF5, C6orf89, MOCS1, SAA1, NMT2, SLC39A7, SIRT5, C9, MRPL2
AC025265.1	− 0.56836	5.46E−05	0.014907	NT5DC3, MTERF2, OVGP1, GOLGA8B, RPL9, SLC25A16, KLHL23, NR2C1, NSUN6, MPST, CENPV, C12orf73, ZNF577, ABCA5, CHKA
ATP1A1-AS1	− 0.42986	0.000822	0.042653	ABCD3, PRKAA2, NBR1, TOM1L1, CNNM3, C16orf58, C1orf56, SPATA25, DDAH1, USP30, CRYZ, ST3GAL3, PARD3B, REPIN1, COX11
EIF3J-DT	− 0.40662	0.000201	0.025372	C2orf15, ZBTB26, VPS39, ZNF512, POLR2M, ETAA1, ZBTB14, HNRNPA1L2, ZNF33B, ICE2, MKS1, ZNF248, KAT8, INTS14, CTDSPL2
AC114956.2	− 0.51268	0.000145	0.021664	C5orf34, NIPBL, ZNF131, RAD1, DROSHA, C5orf51, RICTOR, C5orf22, NUP155, TMEM267, DNAJC21, CPLANE1, ICE1, MARCHF6, PAIP1

### Establishing and validating the 3 lncRNAs based prognostic signature in the training set

**Figure 3:**
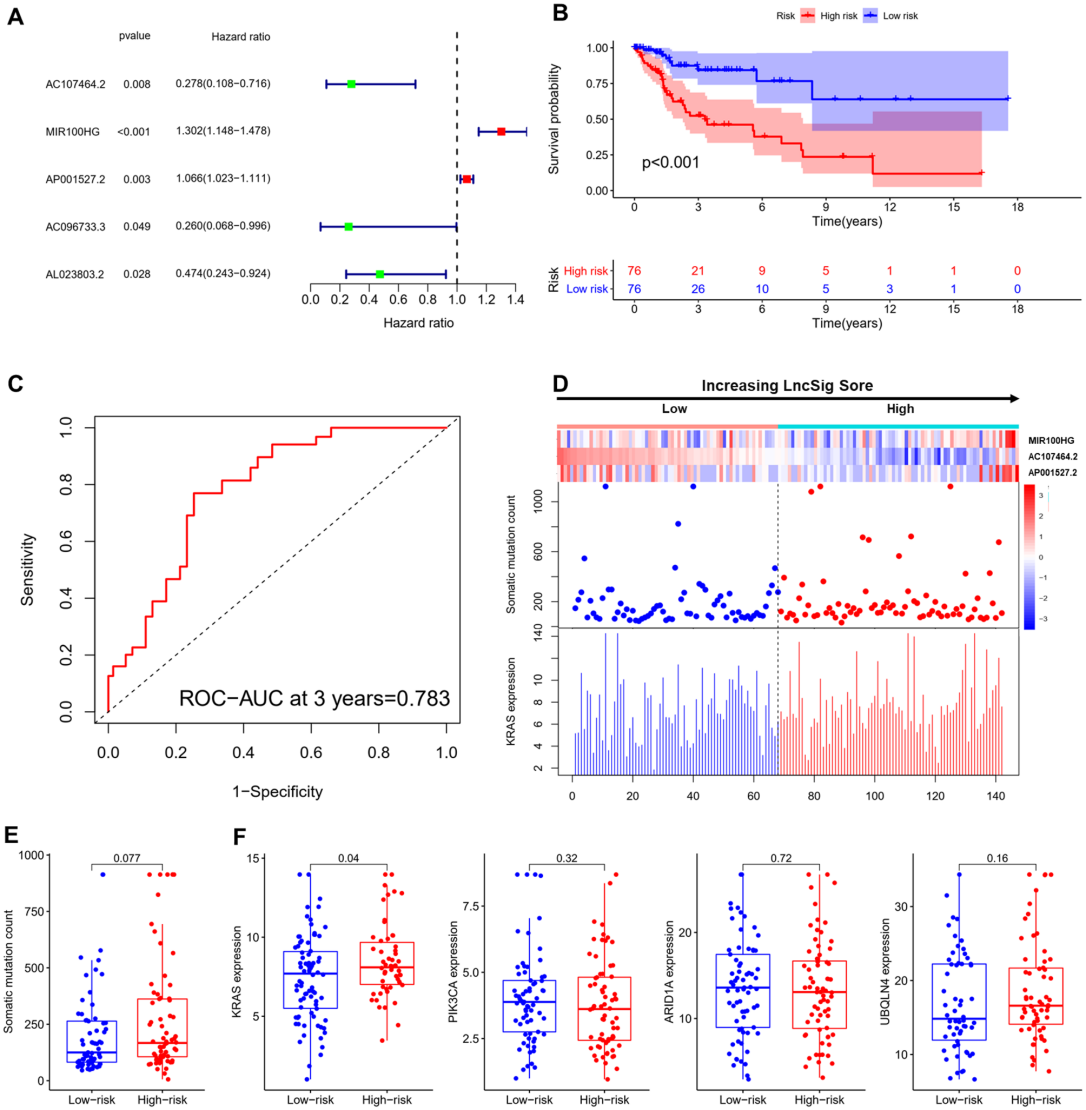
Establishment of the prognostic model and validation of the genomic instability-derived lncRNA signature (LncSig) for outcome prediction in the training set. (**A**) 5 lncRNAs for establishment of the prognostic model. (**B**) Estimates of overall survival of patients with low or high risk predicted by the LncSig in the training set (*p* < 0.001). (**C**) Time-dependent ROC curves analysis of the LncSig at 3 years (AUC = 0.783). (D) With increasing LncSig score, LncRNA expression patterns, the distribution of somatic mutation and KRAS expression. (**E**) The distribution of somatic cumulative mutations in high- and low-risk groups. (**F**) KRAS, PIK3CA, ARID1A and UBQLN4 expression in the high- and low-risk groups. The red represents the high-risk group, and the blue represents the low-risk group.

The prognostic model was constructed by a group of 304 patients with a survival duration of more than 1 month and CC-related genes. The R package caret may randomly separate the whole data set into a training set (n = 152) and a testing set (n = 152). The baseline features are summarized in Table 1. The clinical parameters were not significantly different from the training set and testing set. The univariate Cox proportional hazard regression analysis study 35 genomic instability-associated lncRNAs was then used to establish the 5 candidate lncRNAs prognostic signature (R-package: survival, caret, glmnet, survminer and timeROC, Fig. 3A). After analyzing the training set using the Cox model, we found 3 of 5 candidate lncRNAs (AP001527.2, AC107464.2, and MIR100HG) as independent prognostic lncRNAs in the (*p* < 0.05). The genomic instability-derived lncRNA signature (LncSig) was constructed as follows: LncSig score = (− 1.4997 × expression level of AC107464.2) + (0.3111 × expression level of MIR100HG) + (0.0802 × expression level of AP001527.2). In this LncSig score, positive coef of AP001527.2 and MIR100HG suggested that they might be risk factors for a poor prognosis, while negative ceof of AC107464.2 indicated that it could be a protective factor for survival.

The median risk score (1.1467) was used to divide the training set into the high-risk and low-risk groups based on the LncSig. Kaplan–Meier analysis showed that the survival outcomes of patients in the low-risk group are significantly better than patients in the high-risk group (median survival 1.633 years versus 1.323 years, *p* < 0.001, log-rank test; R-package: survival and survminer, Fig. 3B). The survival rate of the high-risk group was 13.8% at 3 years and that of the low-risk group was 17.1%. The time-dependent ROC curves analysis of the LncSig yielded an area under curve (AUC) of 0.783 at 3 years (R-package: survival, survminer and timeROC, Fig. 3C). As the LncSig score increased, we observed how the count of somatic mutations and an increase in the expression level of KRAS. For the high score group, the expression levels of risk factors (AP001527.2 and MIR100HG) were upregulated, while the expression level of protective factor (AC107464.2) was downregulated in the low score group. Conversely, the low score group held an opposite expression of 3 lncRNAs (R-package: limma and pheatmap, Fig. 3D). Compared with the low-risk group, the somatic mutation was found to be substantially greater in the high-risk group (median 166.5 versus 177, *p* = 0.077, Mann–Whitney U test, R-package: limma and ggpubr, Fig. 3E). The expression levels of newly identified drivers of genomic instability (KRAS, PIK3CA, ARID1A, and UBQLN4) were analyzed, in which KRAS in the high-risk group was significantly higher compared to that of patients in the low-risk group (median 7.221 versus 7.036, *p* = 0.04, Mann–Whitney U test, Fig. 3F). Other divers revealed no significant differences.

### Independent validation of LncSig in the testing set and whole set

**Figure 4:**
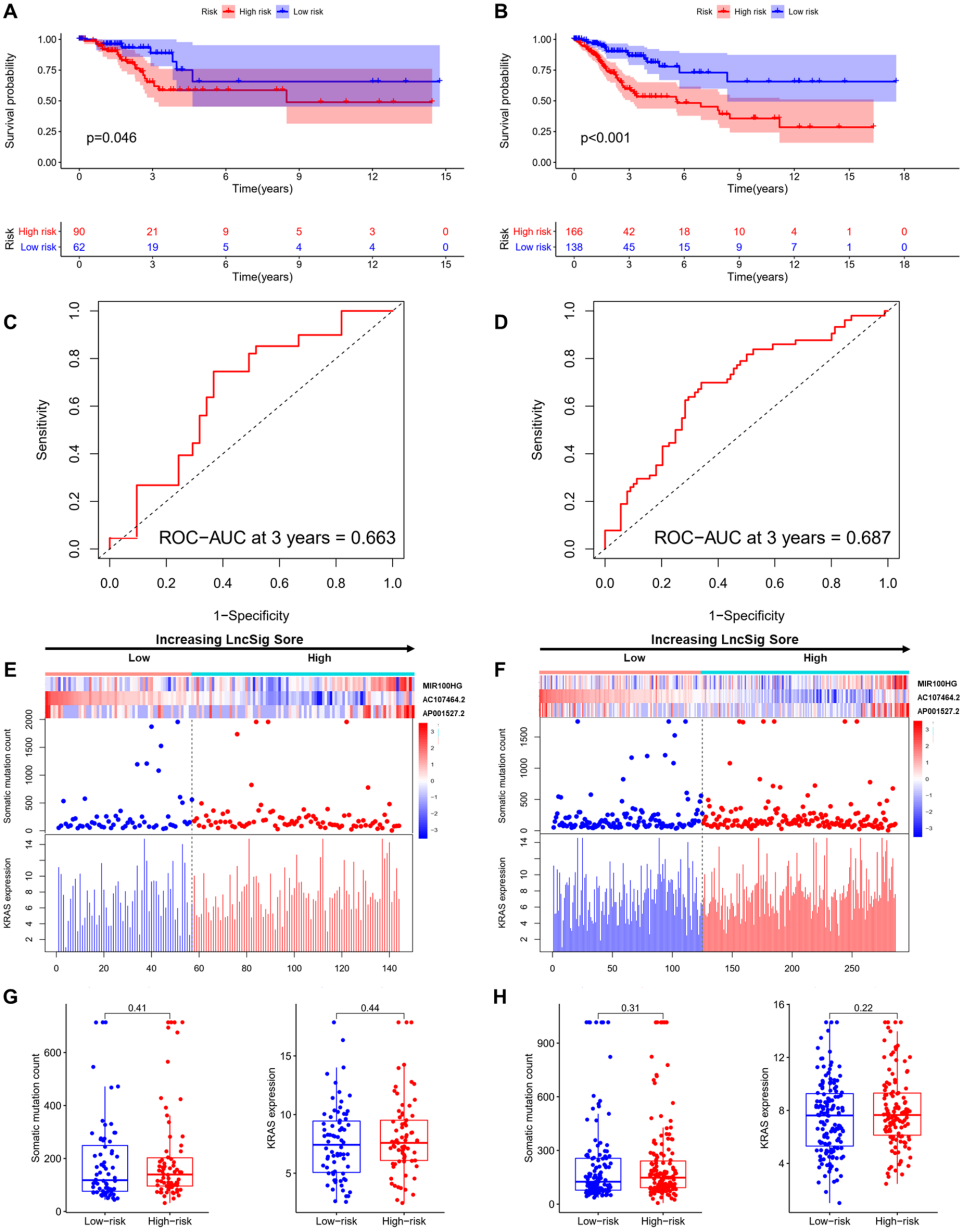
Performance evaluation of the LncSig in the testing and whole set. Kaplan–Meier estimates of overall survival of patients with low or high risk predicted by the LncSig in the testing set (**A**) and whole set (**B**). Time-dependent ROC curves analysis of the LncSig at 3 years in the testing set (**C**) and whole set (**D**). LncRNA expression patterns and the distribution of somatic mutation count distribution and KRAS expression for patients in high- and low-risk groups in the testing set (**E**) and whole set (**F**). The distribution of somatic mutation and KRAS expression in patients of high- and low-risk groups in the testing set (**G**) and whole set (**H**).

To examine the applicability of the LncSig, the testing set (152 patients) was tested for its prognostic outcome in LncSig. The 152 patients of the testing set were assigned to the high-risk group (n = 90) and low-risk group (n = 62) by applying the median risk score (1.1467) of the training set, and the survival rate was significantly different in the testing set (*p* = 0.046). Kaplan–Meier analysis showed that the survival outcomes of patients in the low-risk group are significantly better than patients in the high-risk group (median survival 1.737 years versus 1.611 years, *p* = 0.046, log-rank test; Fig. 4A). The survival rate of the high-risk group was 12.5% at 5 years and that of the low-risk group was 13.8% in the training set. In comparison, the validation was identical to the findings above in the whole set. The patients of the whole set were categorized as the high-risk group (n = 166) and low-risk group (n = 138), which was much higher than patients in the high-risk population median results in the low-risk groups (survival 1.701 years versus 1.485 years, *p* < 0.001, log-rank test; Fig. 4B). The survival rate was 13.8% in the high-risk group at 5 years below 14.8% in the low-risk group.

The time-dependent ROC curves analysis of the LncSig was applied to the testing set yielded an AUC of 0.663 at 3 years (Fig. 4C). The consistent results of time-dependent ROC curves analysis in the whole set were observed as above, an AUC of 0.687 at 3 years (Fig. 4D).

We verified how the count of somatic mutations and expression of KRAS with increasing LncSig score in the testing set and whole set. The distribution of somatic mutation count and KRAS expression in the testing and whole samples were illustrated in Fig. 4E,F. The results of 2 sets were consistent with our earlier research of the training set. The somatic mutation pattern of the high-risk was marginally significantly higher than the low-risk group in the testing set (median 158 versus 146, *p* = 0.41). The expression level of KRAS was observed to be marginally significantly higher in the high-risk group than that in the low-risk group (median 7.469 versus 7.212, *p* = 0.44, *p* = 0.084, Mann–Whitney U test; Fig. 4G). The somatic mutation pattern of the high-risk was marginally significantly higher than the low-risk group in the testing set (median 149 versus 146, *p* = 0.31). The expression level of KRAS in the high-risk group was observed to be marginally significantly higher than that in the low-risk group (median 7.615 versus 7.605, *p* = 0.22, Mann–Whitney U test; Fig. 4H).

### The LncSig model validation of different clinical groups

**Figure 5:**
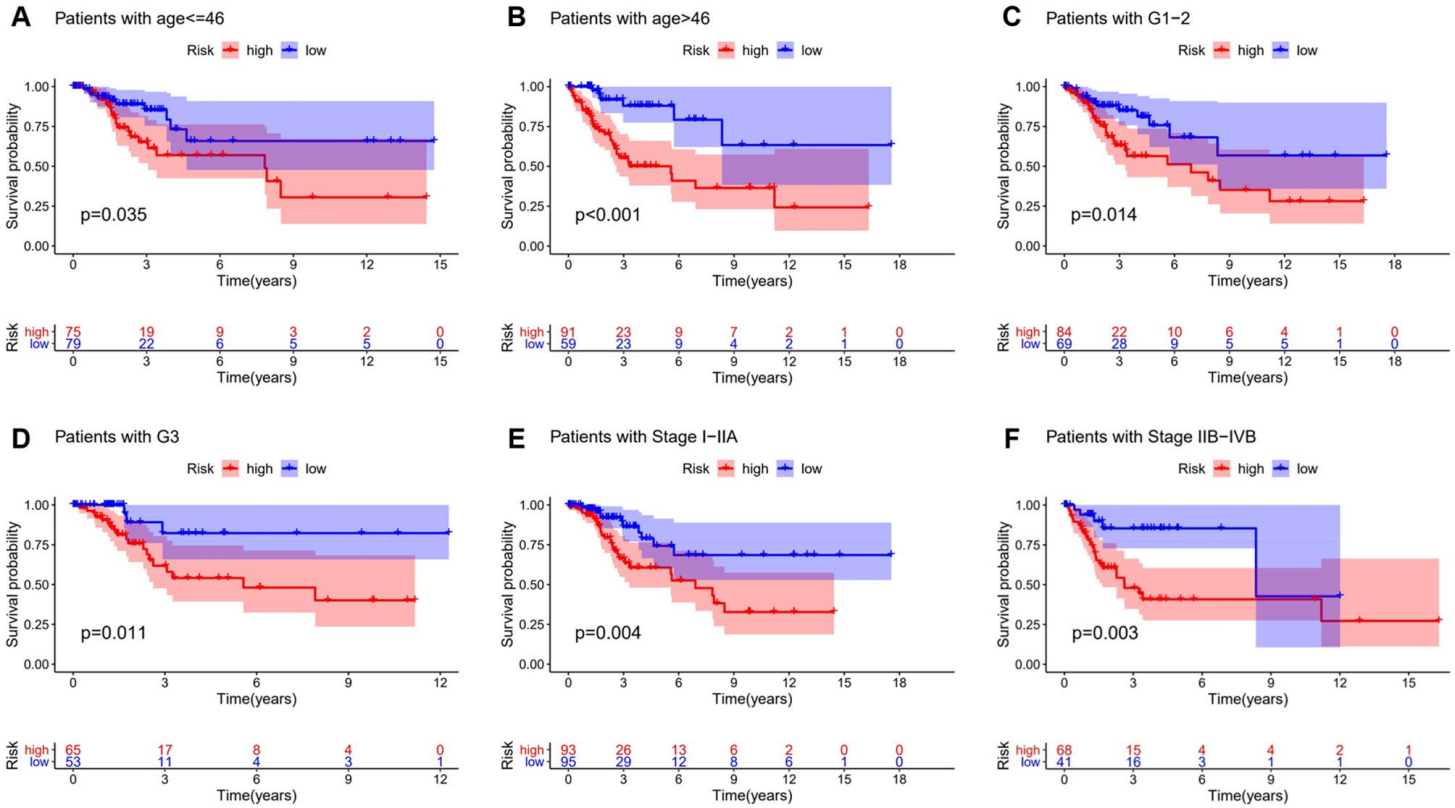
Stratification analyses by age, histological grade and FIGO stage. Kaplan–Meier curve analysis of overall survival in high-risk and low-risk groups for younger patients (age ≤ 46) (**A**) and older patients (age > 46) (**B**). For early-grade patients (histological grade 1–2) (**C**) and late-grade patients (histological grade 3) (**D**). For early-stage patients (FIGO stage I–IIA) (**E**) and late-stage patients (FIGO stage IIB–IVB) (**F**). Statistical analysis was performed using the log-rank test and univariate Cox analysis.

To observe whether the LncSig model was suitable for different clinical groups of patients, we performed multivariate Cox regression analyses on age, histological grade, and FIGO stage. The clinical information table of 3 CC patients set showed that there was no significant difference in age, histological grade, FIGO stage, tumor TNM stage, and vital status between the testing set group and training set group (*p* > 0.05, Chi-square test, Table 1). Stratification analysis was performed to determine whether the LncSig possessed a prognostic value that was independent of the age, histological grade, FIGO stage. Patients in the whole set were stratified into a younger group (n = 154) and an older group (n = 150) according to the median age (46-year-old). Patients in each age group further were divided into the high-risk and the low-risk group by using the LncSig model. There was a significant difference in Kaplan–Meier curve analysis of overall survival between the high-risk and low-risk groups in the younger group (*p* = 0.035, Fig. 5A). There was also a statistical difference in the older group (*p* < 0.001, Fig. 5B). Then patients in the whole set were stratified into a well-moderately differentiated group (histological grade 1–2, n = 153) and a poorly-no differentiated group (histological grade 3, n = 118). LncSig model could further classified patients in each stage into the high-risk and the low-risk group. There was a significant difference between the high-risk and low-risk groups in the well-moderately differentiated histological grade group (*p* = 0.014, Fig. 5C). There was also a statistical difference in the poorly-no differentiated histological grade group (*p* = 0.008, Fig. 5D). Finally, according to different FIGO stages and treatment methods, patients in the whole set were stratified into an earlier stage group (FIGO stage I–IIA, n = 188) and a later stage group (FIGO stage IIB–IVB, n = 109)^14^. LncSig model could further classified patients in each stage into the high-risk and the low-risk group. There was a significant difference between the high-risk and low-risk groups in the earlier stage group (*p* = 0.001, Fig. 5E). There was also a statistical difference in the advanced group (*p* = 0.017, Fig. 5F). The results suggested that the LncSig model was an independent prognostic factor for overall survival in CC patients.

### The prediction outcome of LncSig model greater than KRAS mutation status

**Figure 6:**
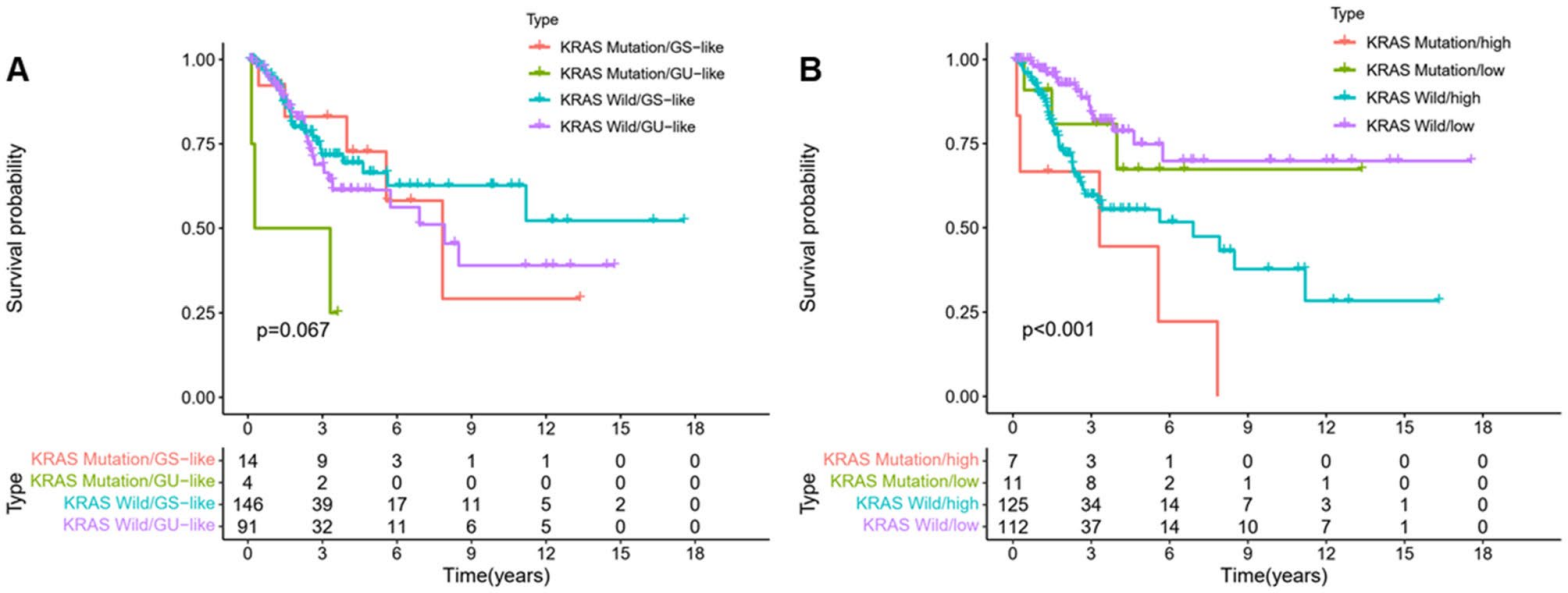
Combined survival analysis of genotyping and mutation. (**A**) Kaplan–Meier curve analysis of overall survival is shown for patients classified according to KRAS mutation status and the GU/GS. (**B**) Kaplan–Meier curve analysis of overall survival is shown for patients classified according to KRAS mutation status and the LncSig.

To further verify the reliability of the LncSig model, we compared it with KRAS mutation status. Samples were classified into the wild group and the mutation group according to their KRAS mutation. We further classified the mutation group based on somatic mutations into two groups: GU-like and GS-like. The wild group is the same as above. As shown in Fig. 6A, the groups were divided into KRAS Mutation/GS-like, KRAS Mutation/GU-like, KRAS Wild/GS-like, and KRAS Wild/GU-like group. The overall survival outcome of KRAS Mutation was lower than that of KRAS wild, R-package: survival and survminer. The result indicated that KRAS mutation/GU-like patients had marginally shorter survival than those with KRAS wild type (*p* = 0.067, log-rank test). According to LncSig, the mutation/wild KRAS group samples were divided into two groups: the high-risk and low-risk. As shown in Fig. 6B, the overall survival outcome of KRAS Mutation/high had significantly lower than those with KRAS wild type (*p* < 0.001, log-rank test). The survival curve of the KRAS Mutation/GU-like group (Fig. 6A) was not similar to KRAS Mutation/high group curves (Fig. 6B). Our results provide a more detailed analysis of the prognosis of patients with KRAS mutations. Therefore, The significant difference suggested that the LncSig may be better than the KRAS mutation status alone.

### Survival performance prediction comparison of the LncSig with existing lncRNA-related signatures

**Figure 7:**
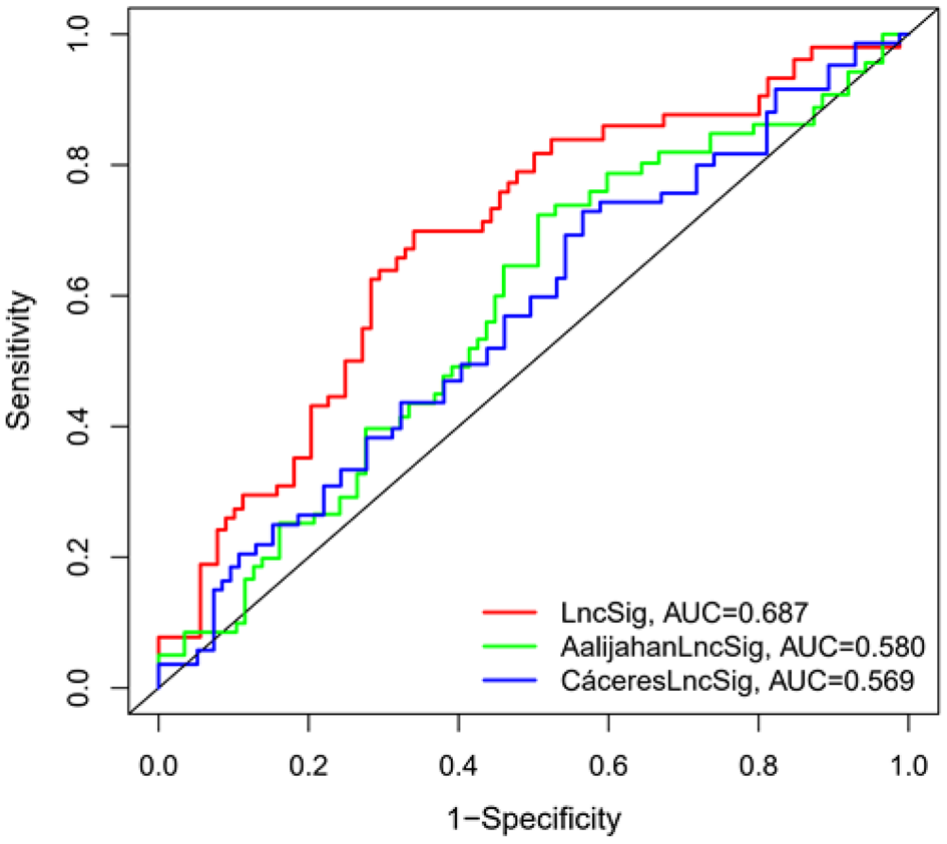
Combined survival analysis of model comparison. The ROC analysis at 3 years of overall survival for the LncSig, AalijahanLncSig and MiguelLncSig.

We further compared the prediction performance of the LncSig with two recently published lncRNA signatures: 3-lncRNAs (H19, MALAT1, and CCHE1) signature derived from Cáceres’ study (hereinafter referred to as CácereslncSig)^15^ and 2-lncRNAs (HOTAIR and SNHG1) signature derived from Aalijahan’s study (hereinafter referred to as AalijahanlncSig)^16^ using the same TCGA patient cohort. As shown in Fig. 7, the AUC at 3 years for the LncSig is 0.687, which is significantly higher than that of CácereslncSig (AUC = 0.569) and AalijahanlncSig (AUC = 0.580), R-package: limma, survival, survminer and timeROC. These comparison results of ROC survival prediction demonstrated the better prognostic performance of the LncSig in predicting survival than two recently published lncRNA signatures.

## Discussion

Cervical cancer is thought to bring a great threat to current women's health, and have important impacts on it. Statistics show that the age of diagnosed patients is tardily decreasing, with 80% developing aggressive cancer. Though traditional tumor grade and pathologic stage are used as the most important prognostic factors in the CC patients, it is still difficult to predict the clinical outcome more accurately^17,18^. However, reliable and specific biomarkers for the diagnosis and prognosis of cervical cancer are scarce and lack exploration. Earlier research had focused on a single biomarker, which might reduce the prognostic performance^19–21^. Therefore, more reliable prognostic models for CC patients currently are urgently need.

Recently, more and more scholars have been drawn to genomic instability. Genomic instability can not only initiate cancer, augment progression, and influence the overall prognosis of the affected patient, but also the survival of CC patients^3,5,22,23^. Recent studies have shown that epigenetic modifications and DNA damage from endogenous and exogenous sources could affect genomic instability^24–27^. An increasing number of reports have revealed that lncRNAs are implicated in the control of various cancer cellular disease progression^28–30^. Though the comprehension of functional mechanisms of lncRNAs has shown that lncRNAs also are crucial for genomic stability, the systematic exploration of genomic instability-associated lncRNAs on their clinical significance in cancers is still in its infancy. Accumulative evidence has identified lncRNAs as functional regulators of cervical cancer oncogenesis and progression, and play critical roles in the regulation of the complex cellular comportements^31–33^. We used a mutator hypothesis-derived computational model, which combined lncRNAs expression profiles and somatic mutation profiles in a tumor genome for screening lncRNAs.

A five-lncRNAs signature based on the TCGA database has been identified and validated in this report. And then, with GO enrichment, KEGG pathway, and co-expression analysis, we explored the potential mechanism of 35 lncRNAs. Our studies suggested that the genes that co-expressed with the 35 lncRNAs were enriched in rRNA catabolic process, deoxyribonucleotide catabolic process, and transcriptionally active chromatin. rRNA that was essential housekeeping genes found in all organisms can maintain genome integrity^34,35^. Regulation of intracellular deoxynucleoside triphosphate (dNTP) pool is critical to genomic stability and cancer development, and imbalanced deoxyribonucleotide catabolic can lead to genomic instability and cell-cycle progression, thus promoting the proliferation of cancer cells^36^. Specific DNA structures such as R-loops and topoisomerase-induced DNA double-strand break (DSBs) causing genotoxic stress and may lead to genome instability and consequently to cancer in the transcriptional activation^37^. According to KEGG pathway analysis, the 35 lncRNAs were involved in transcriptional misregulation in the cancer pathway, ribosome, which are associated with genomic instability^38–40^.

Furthermore, we examined whether genomic instability-related lncRNAs could allow the prediction of CC patients' outcome, and then resulted in a lncRNA signature (LncSig) including three genomic instability-related lncRNAs (AP001527.2, AC107464.2, and MIR100HG). The whole TCGA clinical set was classified into the high-risk and the low-risk group with significantly different survival in the training set, which was verified on the testing set. After a careful literature search, we found that AP001527.2 was associated with the immune microenvironment of cervical cancer^41^. MIR100HG was associated with promoter methylation of cervical cancer^42,43^. The biological function of lncRNA AC107464.2 has not been reported until now. These validation results in multiple data sets indicated that the LncSig could predict the prognosis and genomic instability of CC patients.

Some studies suggested that activating KRAS mutation was the major oncogenic driver regardless of a specific site of origin^12,44,45^. LncSig found that the expression level of KRAS in the high-risk group was observed to be marginally significantly higher than that in the low-risk group. In different clinical groups, we also found that the LncSig had a significantly different clinical outcome in CC patients. Furthermore, the LncSig could marginally significantly distinguish survival outcomes between KRAS mutation patients and other group patients. KRAS mutation/high patients had significantly shorter survival than those with KRAS wild type. The significant difference suggested that the LncSig may be better than the KRAS mutation status alone. These findings suggested that the prediction outcome of the LncSig model might be greater than the KRAS mutation status.

There are still some limitations that require further study. Although LncSig has been validated in the TCGA data set, it required more independent data sets to verify the LncSig to guarantee its reliability and replicability. The regulatory mechanisms of the genomic instability in CC patients are understood via large numbers of verification experiments.

## Conclusion

In summary, we established a signature model based on 3 genomic instability-associated lncRNAs corrected to evaluate progression and prognosis in CC. The high- and low-risk groups present separate survival states, suggesting the capacity of genomic instability-associated lncRNAs to determine the survival of patients. The LncSig provides a critical approach and resource for further studies examining. We expect the LncSig model to pave the way for further research into the function and resource of lncRNAs, as well as a key approach to customizing individual care decision-making.

## Data Availability

The data used to support the findings of this study are available from the corresponding author upon request. The availability of data and materials is from the TCGA database (https://portal.gdc.cancer.gov/repository).
